# Whole-Transcriptome Sequencing Identifies Novel *IRF2BP2-CDX1* Fusion Gene Brought about by Translocation t(1;5)(q42;q32) in Mesenchymal Chondrosarcoma

**DOI:** 10.1371/journal.pone.0049705

**Published:** 2012-11-21

**Authors:** Kaja B. Nyquist, Ioannis Panagopoulos, Jim Thorsen, Lisbeth Haugom, Ludmila Gorunova, Bodil Bjerkehagen, Alexander Fosså, Marianne Guriby, Torfinn Nome, Ragnhild A. Lothe, Rolf I. Skotheim, Sverre Heim, Francesca Micci

**Affiliations:** 1 Section for Cancer Cytogenetics, Institute for Medical Informatics, The Norwegian Radium Hospital, Oslo University Hospital, Oslo, Norway; 2 Centre for Cancer Biomedicine, University of Oslo, Oslo, Norway; 3 Faculty of Medicine, University of Oslo, Oslo, Norway; 4 Department of Pathology, The Norwegian Radium Hospital, Oslo University Hospital, Oslo, Norway; 5 Department of Oncology, The Norwegian Radium Hospital, Oslo University Hospital, Oslo, Norway; 6 Department of Cancer Prevention, Institute for Cancer Research, The Norwegian Radium Hospital, Oslo University Hospital, Oslo, Norway; The Chinese University of Hong Kong, Hong Kong

## Abstract

Mesenchymal chondrosarcomas (MCs) account for 3–10% of primary chondrosarcomas. The cytogenetic literature includes only ten such tumours with karyotypic information and no specific aberrations have been identified. Using a purely molecular genetic approach a *HEY1-NCOA2* fusion gene was recently detected in 10 of 15 investigated MCs. The fusion probably arises through intrachromosomal rearrangement of chromosome arm 8 q. We report a new case of MC showing a t(1;5)(q42;q32) as the sole karyotypic aberration. Through FISH and whole transcriptome sequencing analysis we found a novel fusion between the *IRF2BP2* gene and the transcription factor *CDX1* gene arising from the translocation. The *IRF2BP2-CDX1* has not formerly been described in human neoplasia. In our hospital’s archives three more cases of MC were found, and we examined them looking for the supposedly more common *HEY1-NCOA2* fusion, finding it in all three tumours but not in the case showing t(1;5) and *IRF2BP2-CDX1* gene fusion. This demonstrates that genetic heterogeneity exists in mesenchymal chondrosarcoma.

## Introduction

The classification of sarcomas describes over 50 different histological subtypes [1]. In approximately 20% of them, recurrent balanced translocations leading to formation of fusion genes were identified [2]. Fusion genes provide diagnostic and sometimes prognostic information on the tumours they characterize and knowledge about them could ultimately lead to new targeted therapies [3].

Mesenchymal chondrosarcomas (MCs) are rare tumours that account for 3–10% of primary chondrosarcomas [1]. Their typical histological appearance includes a biphasic pattern with areas of round primitive mesenchymal cells interrupted by chondroid elements [4]. Most cases are diagnosed in the second and third decade of life and the prognosis is mostly poor, with a 5-year survival rate of about 50% [5]. Adequate surgery is the gold standard for treatment of localized disease [6] and the role of chemotherapy and radiotherapy remains poorly defined [7], [8].

According to the Mitelman Database of Chromosome Aberrations and Gene Fusions in Cancer [9], only ten MCs have been karyotyped and no consistent cytogenetic findings have been described. Recently, however, using a genome-wide exon-resolution expression screen, a fusion between the hairy/enhancer-of-split related with YRPW motif 1 *(HEY1)* gene and the nuclear receptor coactivator 2 (*NCOA2)* gene was detected in 10 out of 15 analysed MCs (67%) [10]. Both genes are located on the long arm of chromosome 8 and so the fusion presumably results from an intrachromosomal rearrangement, probably a deletion (∼9.6 Mb according to the UCSC browser, assembly 2009).

We report the finding of a balanced t(1;5)(q42;q32) as the sole karyotypic abnormality in an MC. The translocation led to a new fusion between the interferon regulatory factor 2 binding protein 2 gene (*IRF2BP2)* and the caudal type homeobox 1 *(CDX1)* gene. Based on the recent report by Wang et al (2012) [10], we also examined archival material from another three MCs we had access to for the presence of the *HEY1-NCOA2* gene fusion, finding it in all three.

## Materials and Methods

### Patient Samples

**Figure 1:**
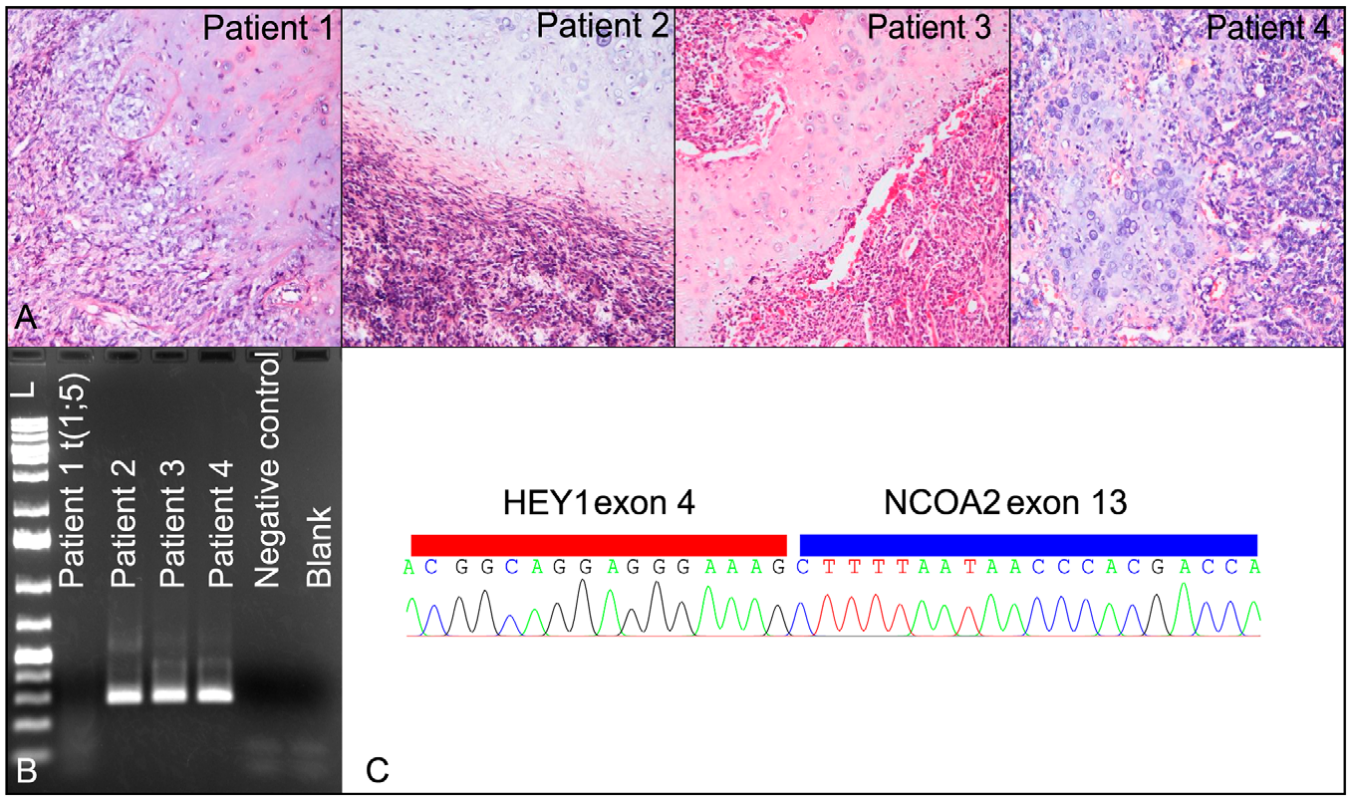
Histological images of the four MCs and characteristics of the *HEY1-NCOA2* fusion. (A) The typical biphasic histological pattern is observed in all tumours. (B) The *HEY1-NCOA2* fusion was detected using primers HEY1_F1 and NCOA2_E13-R3 in patients 2–4 but not in patient 1 whose tumour showed the t(1;5). (C) The *HEY1-NCOA2* fusion was confirmed by sequencing. The breakpoint positions were identical to those previously reported.

Patient 1 was a 63-year-old female in whom a solitary tumour mass was detected in the right cerebral hemisphere in August 2007. Examination of biopsy material revealed the tumour to be a diffuse large B-cell lymphoma of activated B-cell subtype. Cytogenetic analysis of this tumour was unsuccessful. Detailed work-up for other manifestations of lymphoma was negative, compatible with a diagnosis of primary central nervous system lymphoma (PCNSL). However, a tumour in the left iliacus muscle was detected, 3 cm in largest diameter. Biopsies revealed a spindle cell tumour of uncertain malignant potential. The patient received chemotherapy for PCNSL according to Abrey et al. [11] including high-dose methotrexate and high-dose cytarabin. Evaluation after 7 courses of chemotherapy confirmed complete remission of her PCNSL. There was no change in size of the tumour in the left iliac muscle and in June 2008 a wide excision of it was performed. A detailed work up of the tumour specimen revealed a small cell and chondromatous tumour diagnosed as a mesenchymal chondrosarcoma (Figure 1A). Focal infiltrative growth and necroses were present. Because of narrow margins, postoperative radiotherapy 2 Gy × 25 was given. A CNS recurrence of her lymphoma was detected in November 2011, and the patient has received radiation therapy. She remains without sign of recurrence of the MC at the time of writing.

The Norwegian Radium Hospital (NRH) is the largest referral centre for Norwegian patients with bone and soft tissue tumours covering a population of 2.6 million. To identify additional patients with a diagnosis of MC, a database search was performed for cases with this disease. Three additional patients (patients 2–4) were identified (see Table 1 for clinical details and Figure 1A for histological image).

**Table 1:** Patient characteristics.

Patient number	Sex/age at diagnosis	Histological diagnosis	Location of primary tumour	Tumour material analysed
1	F/63	Mesenchymal CS	Left iliacus muscle(soft tissue tumour)	Primary tumour
2	F/38	Mesenchymal CS	Pelvic bone (bone tumour)	Metastasis
3	F/12	Mesenchymal CS	Vertebra (bone tumour)	Primary tumour
4	F/39	Mesenchymal CS	Vertebra and right thigh (bone and soft tissue tumour)	Metastasis

F = female.

### Ethics Statement

Written informed consent was obtained from patients 1 and 3. In the latter case, one of the parents consented on the patient’s behalf. Frozen tissue from deceased patients (patients 2 and 4) was retrieved from The Radium Hospital biobank (project nr S-0747a approved by the Regional Ethics Committee and The Directory of Health (Helsedirektorat) in 2008). Specific permission to perform RNA analysis/sequencing was obtained from patient 1 after approval by the Regional Ethics Committee for Medical and Health Research Ethics South-East (REC number: 2010/1389A). The entire study was also approved by the institutional review board at the Norwegian Radium Hospital. All data were analyzed anonymously.

### G-banding and karyotyping

Fresh tissue from a representative area of the tumour (patient 1) was received and analysed as part of our diagnostic routine. The samples were disaggregated mechanically and enzymatically with collagenase II (Worthington, Freehold, NJ, USA). The resulting cells were cultured and harvested using standard cytogenetic techniques [12]. Chromosome preparations were G-banded with Wright stain. The karyotype was written according to The International System for Human Cytogenetic Nomenclature (ISCN) 2009 guidelines [13]. Phytohemagglutinin (VWR, Oslo, Norway) –stimulated leucocytes were obtained from peripheral blood to determine the patient’s constitutional karyotype.

### Fluorescence in Situ Hybridization Analysis

Fluorescence in situ hybridization (FISH) was performed using probes from bacterial artificial chromosomes (BACs). BACs and fosmid clones flanking and covering the breakpoint positions were selected using the Human Genome Browser at the University of California web site (Feb.2009/release: hg19, http://genome.ucsc.edu/). The selected clones (see Table S1 for detailed information) were purchased from Life Technologies (Carlsbad, CA, USA) or the BACPAC Resource Center (Oakland, CA, USA).

Bacteria were cultured in selective media according to the manufacturer’s recommendation. DNA was extracted using High Pure Plasmid Isolation kit (Roche Applied Science, Penzberg, Germany). DNA labelling was done in a nick translation reaction and the synthesized probes were hybridized to previously G-banded slides. All procedures were performed as previously described [14]. The slides were counterstained with 4′,6-diamidino-2-phenylindole (DAPI). The analysis was done using a CytoVision system (Applied Imaging, Newcastle, UK). All probes were tested for their correct location on normal metaphase spreads prior to use.

### Material for Molecular Analysis

Representative samples of tumour tissue were frozen and stored at −80°C after surgery. DNA was isolated using Genomic-tip (Qiagen, Hilden, Germany) to obtain pure high molecular weight DNA. RNA was extracted from tumour tissue using the Trizol reagent (Life Technologies) with a homogenizer (Omni THQ Digital Tissue Homogenizer, Kennesaw, GA, USA). The RNA quality was evaluated using the Experion Automated Electrophoresis System (Bio-Rad Laboratories, Hercules, CA, USA). cDNA was synthesized using the iScript kit and random primers (Bio-Rad Laboratories). All procedures were done according to the manufacturers’ recommendations.

### High-throughput Paired-end RNA-sequencing

Sequencing was performed according to the TruSeq paired-end RNA-sequencing protocols from Illumina for Solexa sequencing on a Genome Analyzer IIx with paired end module (Illumina Inc., San Diego, CA, USA). 3.5 µg total RNA was used as starting material for library construction, using the TruSeq RNA Sample Preparation Kit v2 where the steps include poly-A mRNA isolation, fragmentation, and cDNA synthesis before adapters are ligated to the products and amplified to a final cDNA library. Shearing to about 150 bp fragments was achieved using divalent cations under elevated temperature. Approximately 58 million clusters were generated by the TruSeq PE Cluster Kit v2 on the Illumina cBot Cluster Generation System, and 76 base pairs were sequenced, from each side of the fragments, using reagents from the TruSeq SBS Kit v5 (all kits from Illumina).

### Gene Fusion Prediction

The Illumina software pipeline was used for processing of image data into raw sequencing data (SCS 2.9 and Casava 1.8.2), and only sequence reads marked as “passed filtering” were used in the downstream data analysis. A total of 91 million reads were obtained. We utilized the fusion discovery software deFuse (version 0.4.3) [15], with Ensembl release 65 reference genome (hg19) and gene models, RepeatMasker, EST, and spliced EST annotations downloaded from the University of California Santa Cruz Table Browser (http://genome.ucsc.edu/, accessed May 2012). UniGene clusters were downloaded from National Center for Biotechnology Information (http://www.ncbi.nlm.nih.gov/, accessed May 2012) to assist in locating potential gene fusions. Three spanning reads and two split reads were required to call sequence reads a gene fusion.

### PCR and Sequencing

Primers used in PCR were designed with the FastPCR software [16]. The full list of applied primers is given in Table 2. The primers used for detection of the *HEY1*- *NCOA2* fusion were identical to the primers used by Wang et al [10]. cDNA PCR was run using 2 µl cDNA in a 25 µl PCR reaction using TaKaRa Ex Taq Hot Start (Takara Bio Inc, Shiga, Japan). The PCR conditions were as follows: 98°C for 7 sec, 68°C for 2 min after a 1 min initial denaturation at 98°C. 34 cycles were run. Amplified products were cloned using the TOPO TA cloning kit (Life Technologies). Selected products were sent for Sanger sequencing (GATC Biotech, Konstanz, Germany) and obtained sequences were analysed using BLAST (Basic Local Alignment Search Tool, www.ncbi.nlm.nih.gov/BLAST/). All cases were tested for expression of a zinc-finger gene suppressor of zeste 12 homolog (Drosophila) (*SUZ12*) to assess RNA quality.

**Table 2:** List of primers.

Primers	Sequence (5′ to 3′)	Tested fusion
HEY1_F1	CGAGGTGGAGAAGGAGAGTG	*HEY1-NCOA2*
NCOA2_E13-R3	AGTTGGGCTTTGCAATGTGA	*HEY1-NCOA2*
CDX1-214F	CCGCAGTACCCCGACTTCTCCAG	*CDX1-IRF2BP2*
CDX1-369F	ATTCGGGCCCCCTCCAGACTTTA	*CDX1-IRF2BP2*
CDX1-659R	GTTCAGTGAGCCCCAGATTGGCAG	*IRF2BP2- CDX1*
CDX1-771R	TGATGTCGTGGGCCATCGGC	*IRF2BP2- CDX1*
CDX1-26970R	GTCTCAGGCTCCCCTTCGTGAGTGTGTC	*IRF2BP2- CDX1*
IRF2BP2-895F	CAAGAGCCGCGGGTCTGGAGA	*IRF2BP2- CDX1*
IRF2BP2-926F	GTCAACAGGCCCAAGACCGTGC	*IRF2BP2- CDX1*
IRF2BP2-1172R	CTTGAGCCCCTCTGTGGATGTGGA	*CDX1-IRF2BP2*
IRF2BP2-1248R	GTGTGGTCCGGTTGGAATGAGGTG	*CDX1-IRF2BP2*

### Long Distance PCR

PCR experiments on genomic DNA were performed using ∼100 ng DNA as template in 25 µl PCR reactions using TaKaRa LA Taq following the manufacturer’s recommendations for LD-PCR: 30 cycles of 98°C for 10 sec (denaturation) followed by 68°C for 15 min (annealing and extension; Takara Bio Inc). PCR products were purified using GeneJET PCR purification kit (Fermentas GmbH, St. Leon-Rot, Germany) and sent for Sanger sequencing (GATC Biotech).

## Results

**Figure 2:**
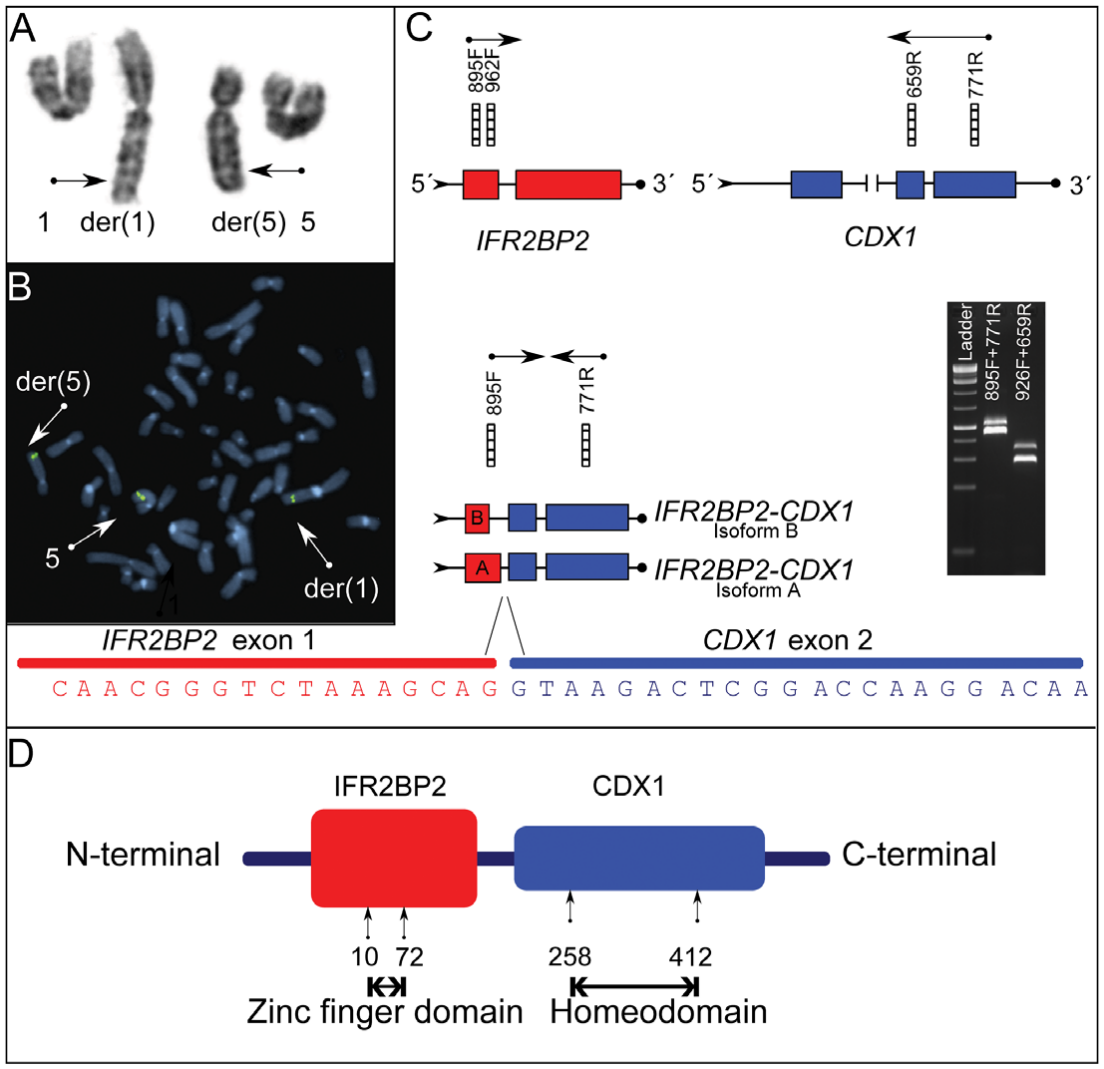
Cytogenetic and molecular details of the *IRF2BP2- CDX1* fusion gene. (A) Partial karyotype showing the aberrant chromosomes 1 and 5. Arrows point to the breakpoint positions. (B) Inverted DAPI metaphase harbouring the t(1;5). Upon hybridization with probe CTC-802J2 mapping to 5q32, three fluorescent signals were detected; (on the normal chromosome 5, the derivative chromosome 5, and the derivative chromosome 1), indicating a breakpoint within the genomic area covered by this BAC. (C) In the upper panel, the structure of the wild type *IRF2BP2* and *CDX1* genes is shown in grey and black, respectively. Bars indicate positions of primers yielding products by cDNA PCR. For detailed primer information, see Table 2. In the lower panel, the two identified fusion gene transcripts are illustrated. By sequencing the fusion was found to consist of *IRF2BP2* exon 1 (isoform A or B) fused to exon 2 of *CDX1*. The base sequence shown originates from isoform A. A gel blot demonstrating the two PCR products is shown in the right panel. The primer combinations used are specified. (D) Illustration of hypothetical fusion protein. The N` terminal part of the protein originates from IRF2BP2 and harbour a zinc finger motif which may bind DNA [17]. The C` terminal part contains a homeodomain which may also interact with the DNA double helix (http://www.ncbi.nlm.nih.gov/gene/1044, accessed August 2012).

The cytogenetic analysis of the only tumour (patient 1) from which we got a fresh sample revealed a balanced t(1;5)(q42;q32) as the sole abnormality in all cells analysed (Figure 2A). Analysis of peripheral blood leukocytes displayed a normal female karyotype ruling out the possibility of a constitutional aberration.

All cases (i.e., the tumour carrying a t(1;5) as well as the three archival tumours) were tested for the *HEY1-NCOA2* fusion which was recently identified in 10 of 15 investigated MCs [10]. A PCR product of approximately 300 bp was amplified from tumours 2, 3, and 4 but not from tumour 1. Subsequent sequencing analysis confirmed the fusion between *HEY1* exon 4 and *NCOA2* exon 13 in cases 2–4, identical to the one previously described [10] (Figures 1B and 1C). As no PCR product was amplified in tumour 1, i.e., the one showing the t(1;5), we assumed that a new fusion gene was generated by the 1;5-rearrangement. To better characterize the breakpoint on the rearranged chromosome 5, a series of selected BAC clones mapping to the involved bands were hybridized to metaphase plates. Clone CTC-802J2 mapping on 5q32 and covering four genes gave three signals on metaphase chromosomes. The breakpoint position was further narrowed down using fosmid clones with clone G248P81640F4 giving a split signal, mapping the breakpoint to a genomic area between the 3′end of the platelet derived growth factor receptor β gene (*PDGFRβ*) and the large intron 1 of *CDX1.*

Since the breakpoint region as delimited by FISH was large, probably involving one of two genes, we decided to investigate the translocation in detail using a whole transcriptome sequencing approach focusing on potential fusion transcripts between chromosomes 1 and 5. The deFuse algorithm [15], designed for fusion gene discovery in paired-end RNA sequence data sets, gave us a list of 92 putative fusions in the tumour transcriptome that we reduced to 85 after removing isoforms of the same fusions (Table S2). An *IRF2BP2-CDX1* transcript involving two coding regions yielded the highest split count value (number of split reads supporting the fusion) of all the predicted fusions and was predicted to be in-frame. We therefore chose to focus on this putative fusion. cDNA PCR experiments with specific primers were run to validate the *IRF2BP2-CDX1* fusion. Two distinct bands were identified using the primer IRF2BP2-895F, located in exon 1, in combination with CDX1-771R, located in exon 3 (Table 2 and Figure 2C). The primer combination IRF2BP2-926F and CDX1-659R yielded smaller but similar bands. Cloning of the amplified PCR products was performed and sequencing was carried out from six individual bacterial clones. *IRF2BP2* exists as two different isoforms where isoform B lacks 48 bp of exon 1 sequence representing 16 amino acids [17]. Sequencing analysis of the PCR products confirmed the presence of both isoforms fused to exon 2 of *CDX1*, i.e., an *IRF2BP2-CDX1* fusion transcript was confirmed in the tumour RNA. Both fusion transcripts were found to be “in frame” and are predicted to encode proteins of 466 and 450 amino acids, respectively, before being terminated by a stop codon. The reciprocal fusion between *CDX1-IRF2BP2* did not yield any products by cDNA PCR (primer combinations CDX1-214F+IRF2BP2-1248R and CDX1-369F+IRF2BP2-1172R, Table 2). The presence of the *IRF2BP2-CDX1* fusion gene was tested for in specimens from tumour 2–4 using primer combinations IRF2BP2-926F and CDX1-771R (Table 2). None of the specimens showed such fusion.

Next, we wanted to identify the genomic breakpoints of the *IRF2BP2-CDX1* fusion. Using primers IRF2BP2-926F and CDX1-26970R (Table 2) we managed to amplify a product of about 800 bp which by sequencing was shown to contain the breakpoint, i.e., sequencing analysis confirmed the predicted positions of the genomic breakpoints. On chromosome 1 the breakpoint was in intron 1 of *IRF2BP2* (chr1∶234743757 bp), whereas on chromosome 5 it was in the large intron 1 of *CDX1* (chr5∶149551799 bp).

To investigate the involvement of the *IRF2BP2* gene also by FISH, we hybridized BAC clones overlapping *IRF2BP2* to metaphases obtained from the cultured cells (Table S1). Signals were detected on the normal chromosome 1 and the derivative chromosome 1. However, no signal was seen on chromosome 5 as would be expected if the *IRF2BP2-CDX1* fusion had resulted from a simple balanced translocation. These findings thus indicate that a more complex rearrangement had taken place, possibly including inversions at the breakpoint.

## Discussion

The cytogenetic knowledge on mesenchymal chondrosarcomas is limited to ten cases [9]. We report here a solitary t(1;5)(q42;q32) in a case of MC. The translocation led to recombination of the *IRF2BP2* and *CDX1* genes.

This is the first time an *IRF2BP2-CDX1* fusion has been detected in human neoplasia. *CDX1* belongs to the homeobox gene family [18]. These genes share a homebox domain that encodes a DNA binding protein functioning as a transcription factor [19]. In particular, CDX1 is an upstream regulator of Hox-gene expression [20] that has been implicated in malignancies such as leukaemias [21], [22]. In adults, CDX1 expression is restricted to intestinal epithelium [23]–[25] and aberrant expression has been linked to intestinal cancer [24], [26]–[28]. No fusion gene involving *CDX1* has so far been described as opposed to another member of the Cdx family, *CDX2. CDX2* is overexpressed in both lymphoid and myeloid leukaemias [29]–[31] and a fusion gene resulting from a balanced t(12;13) leading to an *ETV6-CDX2* fusion was detected in a patient with acute myeloid leukaemia [32].

The first exon of *IRF2BP2* forms the 5`end of the *IRF2BP2-CDX1* fusion. *IRF2BP2* normally exists in two isoforms resulting from alternative splicing of the gene [17]. Both variants contain a Zinc finger motif at their N-terminus possibly binding DNA [17]. Although no direct link to cancer has been described for this gene, *IRF2BP2* interacts with partners that are involved in cancer as for example the tumour suppressor gene *TP53* [33] and the oncogene *IRF2* [17]. *IRF2BP2* also acts as a co-repressor of *IRF2*, inhibiting the expression of interferon-responsive genes. Recently also *NFAT1*, which encodes a transcription factor involved in the cell cycle, differentiation, and apoptosis, was shown to be repressed by *IRF2BP2* [34]. According to BioGPS [35], *IRF2BP2* is expressed in a variety of human tissues [36].

Two PCR products were obtained by cDNA PCR investigations for the *IRF2BP2-CDX1* fusion. The difference between the two products was by sequencing shown to be caused by the alternative splice variants of *IRF2BP2*. Both sequences were shown to be in frame, with the largest transcript predicted to encode a 466 amino acid protein and the smaller encoding 450 amino acids. The biological implications of the predicted fusion protein IRF2BP2-CDX1 can only be speculated upon, but as both fusion partners are involved in transcriptional regulation, a protein disturbing DNA transcription is likely. The *IRF2BP2-CDX1* fusion is thus suggested to take part in MC tumorigenesis and/or progression.

MCs are rare, and at our institution only five patients (four included in this study) received this diagnosis during the last 25 years. We identified the *HEY1-NCOA2* in three of these tumours (patients 2, 3, and 4), confirming that this fusion gene is common in MC. In a majority of the well-known translocation-related sarcomas such as myxoid liposarcoma and low-grade fibromyxoid sarcoma, more than one defining fusion variant has been detected. Often one fusion variant is more common than the others [2], [3]. *IRF2BP2-CDX1* could be such an additional fusion variant identified in a subset of MCs, but only analysis of larger series of tumours can determine the prevalence of the *IRF2BP2-CDX1*.

Of possible interest is the fact that the three *HEY1-NCOA2*-positive MCs all had tumour manifestations detected in bone, whereas the MC showing the t(1;5) and *IRF2BP2-CDX1* fusion originated from soft tissue. Although most common in bone, one-fifth to one-third of MCs do arise in soft tissue [4]. The tissue of manifestation was not reported in the MCs where the *HEY1-NCOA2* was first described [10]. Given the rarity of these tumours, only future surveys of larger groups of patients can clarify if there is a correlation between the tissue the tumour affects and the type of fusion gene present. This study demonstrates the feasibility and indeed advantage of using karyotyping and molecular cytogenetic methods together with transcriptome sequencing to identify fusion genes caused by chromosomal rearrangements. Traditionally, chromosome walking using BACs or equivalent probes has been used to narrow down the breakpoint regions followed by PCR based analyses to amplify the genes involved in the breakpoints. Submicroscopic rearrangements in the breakpoint area can cause considerable confusion, however, and prevent amplification of fusion genes. Using whole-transcriptome sequencing without prior genetic knowledge of the tumour investigated can also be challenging as validation of numerous predicted fusion gene transcripts is necessary. To know which chromosomes take part in the rearrangement therefore helps considerably when looking for novel putative cancer-specific fusion genes.

## Supporting Information

Table S1 **BAC probes used for FISH experiments in case 1.**
(XLS)

Table S2 **List of fusions suggested by the deFuse algorithm.**
(XLS)
